# Evolution of RAS Mutational Status in Liquid Biopsies During First-Line Chemotherapy for Metastatic Colorectal Cancer

**DOI:** 10.3389/fonc.2020.01115

**Published:** 2020-07-16

**Authors:** Susanne Klein-Scory, Ingo Wahner, Marina Maslova, Yosef Al-Sewaidi, Michael Pohl, Thomas Mika, Swetlana Ladigan, Roland Schroers, Alexander Baraniskin

**Affiliations:** ^1^IMBL Medical Clinic, Ruhr University Bochum, University Hospital Knappschaftskrankenhaus Bochum GmbH, Bochum, Germany; ^2^Department of Radiology, University Hospital Knappschaftskrankenhaus Bochum GmbH, Ruhr University Bochum, Bochum, Germany; ^3^Department of Medicine, University Hospital Knappschaftskrankenhaus Bochum GmbH, Ruhr University Bochum, Bochum, Germany

**Keywords:** digital droplet PCR, BEAMing, colorectal cancer, clonal selection, neoRAS wild-type, liquid biopsy, methylation WIF1 promotor, circulating tumor DNA

## Abstract

Treatment options for patients with metastatic colorectal cancer (mCRC) are limited. This particularly affects the largest group of patients with RAS mutations, who are considered ineligible for therapy with antiEGFR antibodies. In this liquid biopsy-based study, we performed the first in-depth analysis of the RAS mutational status in initially RAS-mutated patients during first-line therapy. RAS status of twelve patients with initially RAS-mutated mCRC was monitored longitudinally in 69 liquid biopsy samples. We focused on patients with stable disease (SD) or partial remission (PR) during first-line therapy (11 patients). Detection of fragmented RAS-mutated circulating cell-free tumor DNA (ctDNA) in plasma was performed by digital-droplet PCR (ddPCR) and BEAMing. Patients' total tumor masses were determined by measuring the tumor volumes using CT scan data. All patients with PR or SD at first follow-up showed a significant decrease of RAS mutational load. In ten patients (91%), the ctDNA-based RAS mutational status converted to wild-type in ddPCR and BEAMing. Remarkably, conversions were observed early after the first cycle of chemotherapy. Plasma concentration of ctDNA was controlled by determination of methylated WIF1-promotor ctDNA burden as a second tumor marker for mCRC. Persistent presence of methylated WIF1-promotor fragments confirmed the ongoing release of ctDNA during treatment. In patients with initially RAS-mutated mCRC, RAS mutations rapidly disappeared during first-line therapy in liquid biopsy, independent of type and intensity of chemotherapy and irrespective of anti-VEGF treatments. Following our results demonstrating conversion of RAS-mutational status, potential effectiveness of anti-EGFR antibodies in selected patients becomes an attractive hypothesis for future studies.

## Introduction

Colorectal cancer (CRC) is the second leading cause of cancer worldwide with 1.85 million people affected globally and 881,000 deaths annually (1, 2). Approximately 20% of all patients present with *de novo* metastatic disease, and, 30% of patients with stage II/III disease develop relapse within 5 years of initial treatment (3).

Metastatic colorectal cancer (mCRC) requires systemic treatment in the majority of patients. Today, the median overall survival for patients with metastatic disease is limited to ~30 months (4). The main therapy backbones have been unchanged for years and comprise anti-epidermal growth factor receptor (EGFR) or anti-vascular endothelial growth factor (VEGF) monoclonal antibodies (mAb), oxaliplatin, irinotecan, and inhibitors of thymidylate synthase activity (4). In nearly 55% of all mCRC patients, RAS-mutated tumors are diagnosed. According to approval and current guidelines, anti-EGFR mAbs are not indicated for the treatment of RAS-mutated mCRC (4–6). Thus, there is currently no approved molecularly targeted treatment for mCRC patients with RAS mutations.

Tumor heterogeneity is hypothesized to play a critical role in the limited prognosis of mCRC and represents a major obstacle to currently applied precision cancer therapy. Basically, the concept of tumor heterogeneity postulates that a single tumor consists of numerous tumor cell subclones, which constantly arise due to genomic mutation. Subclonal diversity offers a selective advantage for cancer cell survival due to the higher probability of preexisting individual resistant clones and thus acquired resistance (6). Selective mechanisms during chemotherapy can result in significant changes of tumor biology (7, 8).

Previously, it has been recognized that RAS mutations can develop during anti-EGFR mAb treatment, and, that these acquired RAS mutations can be predictive for reduced benefit from this therapy (6, 9–14). Interestingly, RAS mutations also can arise without selective mechanisms during tumor development. In this situation, treatment modifications may result in disappearance of RAS-mutated clones (6).

Considering these findings, we studied ctDNA collected form patients with initially RAS-mutated mCRC, the largest mCRC patient group, and asked whether similar selection mechanisms can be observed in this patient group during first-line therapy. May clonal selection lead to the disappearance of the RAS-mutated clones opening new unexpected perspectives for an anti-EGFR mAb therapy for initially RAS-mutated CRC?

Here, we demonstrate that RAS mutations disappear rapidly after the first cycles of chemotherapy. Furthermore, we monitored the dynamic of RAS mutations during therapy.

## Materials and Methods

### Patients, Sample Preparation, and DNA Isolation

The University of Bochum Ethical Committee approved collection and analyses of samples in this study (registration number: 16-5961; ethics committees of Ruhr-University of Bochum). Patients‘written informed consents were obtained prior to sample collection and analysis. Peripheral blood samples were collected in STRECK BCT tubes or in K2-EDTA Vacutainer tubes (Becton Dickenson). After removal of cell debris, plasma samples were stored in aliquots at −80°C until further analyses. The cohort of 12 therapy naïve patients comprised 5 male and 7 female patients with a CRC diagnosis at a median age of 69 years. The plasma volumes for circulating fragmented DNA isolation by QIAamp circulating nucleic acid kit (Qiagen, Hilden, Germany) were 1 ml from initial samples and 3 ml from monitoring samples following the manufacturer's protocol (Qiagen, Hilden, Germany). The elution volumes were adjusted to plasma volumes and ddPCR data were normalized to 1 ml plasma. The fractional abundance measured by ddPCR experiments did not depend on the sample volume used for ctDNA isolation (Supplementary Figure 1A). Further information about the circDNA amounts are given in Supplementary Figures 1B,C.

### Mutation Detection in Tissue Samples

Formalin-fixed and paraffin-embedded tissue sections derived from primary tumors and from metastasized tumor tissue were analyzed by next generation sequencing according to the procedures established for routine clinical use (Institute for Pathology of the Ruhr-University Bochum, Germany). DNA derived from tumor tissues was analyzed for RAS and BRAF mutations.

### RAS Mutation Detection in Plasma Samples

Two different methods to monitor dynamic changes of the mutational status in plasma samples were applied: digital droplet PCR (ddPCR) and BEAMing (OncoBEAM^TM^ RAS CRC CE-IVD kit, Sysmex Inostics GmbH). First, all relevant KRAS variants were monitored using ddPCR assays (KRAS screening G12/13 kit, Bio-Rad Laboratories Inc.). All ddPCR assays were carried out in duplicate according to manufacturer's instructions (Bio-Rad Laboratories Inc.). For each reaction, a standard volume of 20 μl was prepared using 6 μl DNA eluate and 14 μl mastermix (containing 2x supermix without dUTP, 20x specific mutation and wild-type assays from Bio-Rad, and DNA-free water). After droplet generation, amplifications were performed with initial denaturation at 95°C for 10 min, followed by 40 cycles at 94°C for 30 sec and 55°C for 1 min with a ramp rate of 2°C/sec, and, a final inactivation step at 98°C for 10 min.

The definition of wild-type or mutational status depends on the cut-off level of the detection method and this level was dependent on the amount of wild-type DNA. Mutational allele frequencies were calculated as ratios of mutated copies to the sum of wild-type and mutated copies. We set the cut-off of ddPCR-based KRAS mutations at 0.25% mutational frequency (MAF). The ddPCR procedure followed the recommendations of Bio-Rad instructions for rare event detection. The cut-off level of ddPCR KRAS mutational analysis was verified in comparison to BEAMing assays (Supplementary Figures 2A,B).

In samples with no detection of RAS mutations by ddPCR, BEAMing was used to assess the decrease of RAS mutations more accurately and to rule out the potential emergence of new RAS mutations. In addition, NRAS mutations were monitored by BEAMing. The procedure of detecting RAS mutations by BEAMing has been previously described (6, 15, 16) The cut-off to detect a RAS mutation was set at 0.02% as published (9, 15, 16). A comparison of ddPCR and BEAMing KRAS analysis is presented in the Supplementary Figure 2. Our findings confirmed the data of comparative analysis of RAS mutant detection by liquid biopsy assays (17).

### Analyses of Molecular Alterations of Circulating DNA in Plasma Samples

Circulating tumor DNA (ctDNA) samples were tested for PIK3CA and BRAF allele variants. The E545K and H1047R mutant variants of PIK3CA and the V600E mutant variant of BRAF were analyzed using ddPCR assays (for details see Supplementary Table 1). The cut-off for PIK3CA mutant alleles (E545K and H1047R) and the BRAF V600E allele were defined at 0.3% MAF and 0.2% MAF, respectively. None of our circulating DNA samples contained detectable amounts of PIK3CA or BRAF mutated allele variants.

Digital droplet (dd)PCR assays offer the opportunity to quantify ctDNA isolated from plasma (18). Therefore, ddPCR assays (Bio-Rad) were used to monitor the absolute amount of circulating DNA during therapy in order to support the BEAMing assay results in the same plasma sample. The sum of wild-type and mutated copies measured by ddPCR assays was calculated considering the assay volume of ctDNA and plasma sample.

To analyze a possible hematopoetic cell origin of mutated allele fragments in plasma, cellular ingredients of the blood samples were separated by 10 min centrifugation at 1600 × g and careful removal of the overlying plasma. Contamination of the cellular pellet by remaining plasma was estimated at 1%. This contamination of the cellular pellet can result in the mutated allele fragment detection (MAF > 0.25%), if the mutational allele frequencies in plasma exceeded 25%. A 200 μl aliquot of the cellular fraction was used for QIAamp DNA blood Mini Kit (Qiagen GmbH Hilden, Germany) and the genomic DNA of blood cells was isolated following the spin procedure described in the manufacturer's instructions.

### Measurement of WIF1-Promotor Methylation by ddPCR

To confirm the presence of ctDNA at the time point of conversion to neo RAS wild-type, we examined the circulating DNA using the methylation marker WIF1. The promotor hypermethylation in CpG islands is a common epigenetic alteration in colorectal cancer and is observed in 70–90% of cases. Particularly methylation of CpG islands in the WIF1-promotor is considered to be a marker of colorectal cancer (19). Detection of CpG island of WIF1-promotor methylation of circulating DNA in plasma was performed according to the procedure described earlier (20, 21). Briefly, the plasma isolated circulating DNA was modified by bisulfite conversion EZ Lightning kit (Zymo Research). In the methylation specific ddPCR amounts of WIF1-promotor CpG hypermethylated copies were measured in plasma DNA samples at the time of diagnosis and in the earliest available sample after RAS mutational loss (for details see Supplementary File 1B and 6). The relative amount of WIF1-promoter methylation is reported as MAF% vs. the C-LESS-C1 copies (20, 22). C-LESSC1 is a recognized control for absolute DNA amount in the sample (23) (Supplementary File 1B).

### Measurement of Total Tumor Load

Patient total tumor masses were determined by measuring tumor volumes in computertomographies (CT) during disease course.

All CT-based measurements were done on commercial PACS workstations (Jive-X-PACS, Bochum, Germany) by two experienced radiology specialists. The reconstructed slice thickness was 4 mm or less for volumetry of liver and lung metastases.

Scheduled follow-up CT scans were performed every 3 months, after 6 cycles of chemotherapy, or in cases with suspicion of progressive disease, respectively.

### Statistical Analysis

The Fisher exact test (2 × 2 contingency table) with approximation for small sample sizes was performed including all samples of 12 patients. The data were analyzed by unpaired *t*-tests with Welch correction (see Supplementary Material). All statistical analyses were done by Graph path prism 4 software.

## Results

### Patients Characteristics and Treatments

Twelve patients with mCRC and RAS mutations at diagnosis were included into the study. The primary tissue samples (FFPE tumor tissue) of all patients were classified as RAS-mutated and BRAF wild-type by next generation sequencing according to routine molecular-pathologic techniques. All patients had been treated according to national guidelines with FOLFIRI, FOLFOX, FOLFOXIRI, or 5-Fluoruracil (5-FU) with (8 patients) or without bevacizumab (4 patients), respectively (Table 1).

**Table 1:** Patient baseline characteristics and dynamics of mutations in liquid biopsy.

**Patient No. age/sex**	**Sidedness**	**RAS mutant**	**Therapy**	**Meta stases**	**Clinical response**	**Meth AF initial**	**RAS MAF (%)**	**RAS MAF (%)**	**RAS status**	**Time to reach RAS wt**	**RAS wt period**
Age/sex		Codon	Initial	Site	Initial	Initial	Initial	Change	Acquired	w/cyc	Months
1 66/m	R	KR2_12	FOLFOX	hep	PR	nd	1.65	−89.7	wt	8 / 4	n.a.
2 65/f	R	KR2_12	beva+5-FU	hep +PC	PR	12.8	13.7	−100	wt	10 / 6	>4.5
3 65/f	L	KR2_12	FOL FOXIRI	hep	PR	14.6	14.5	−99.2	wt	5 / 3	<1
4 76/f	R	KR2_12	beva+ FOLFOX	hep +PC	PR	0.82	7.8	−96.9	wt	4 / 2	6.5
5 60/f	R	KR2_12	beva+ FOLFOX	hep	PR	10.2	17.5	−98.8	wt	14 / 8	n.a.
6 59/m	R	KR2_13	beva+ FOLFOX	hep +oss	PR	10.3	54	−100	wt	4 / 2	1.5
7 84/m	R	KR2_12	beva+ FOLIRI	hep +pul	PR	10.0	35.4	−100	wt	8 / 4	7
8 66/f	R	KR2_12	beva+ FOLFOX	LN	SD	nd	5.9	−100	wt	4 / 2	15
9 63/m	L	NR3_61	beva+ FOLFOX	hep +pul	PR	nd	12.8	−99.9	wt	24 / 12	-
10 74/f	L	KR2_12	FOLFIRI	hep +oss +LN	PR	22.6	0.48	−60.4	wt	6 / 3	>8.5
11 71/m	L	KR2-13	beva+ FOLFIRI	hep +pul +LN	PR	nd	46	−81	mut	-	-
12 77/m	R	KR2_12	FOLFOX	hep	PD	2	0.3	+167	mut	-	-

*Abbreviations: m, male; f, female, R, right; L, left; beva, bevacizumab; hep, hepatic, pul, pulmonal; PC, peritoneal carcinomatosis; LN, involvement of lymph nodes; oss, osseous metastases; wt, wild-type; mut, mutated; w, weeks; cyc, cycles; mo, month; n.a, not available. Meth AF%, initial WIF1 promotor methylation fraction; RAS MAF, RAS mutational allele frequency*.

At first sampling for liquid biopsy, all patients had newly diagnosed disease and were chemotherapy-naïve. The majority of patients (66.6%) suffered from right-sided colorectal cancer. All but one patient had hepatic metastases (92%), lung metastases were present in 25%. As response to first-line treatment, 10 patients achieved PR, one patient SD, and one patient PD in first follow-up CT scan, respectively.

### Dynamics of RAS Mutation Load

**Figure 1:**
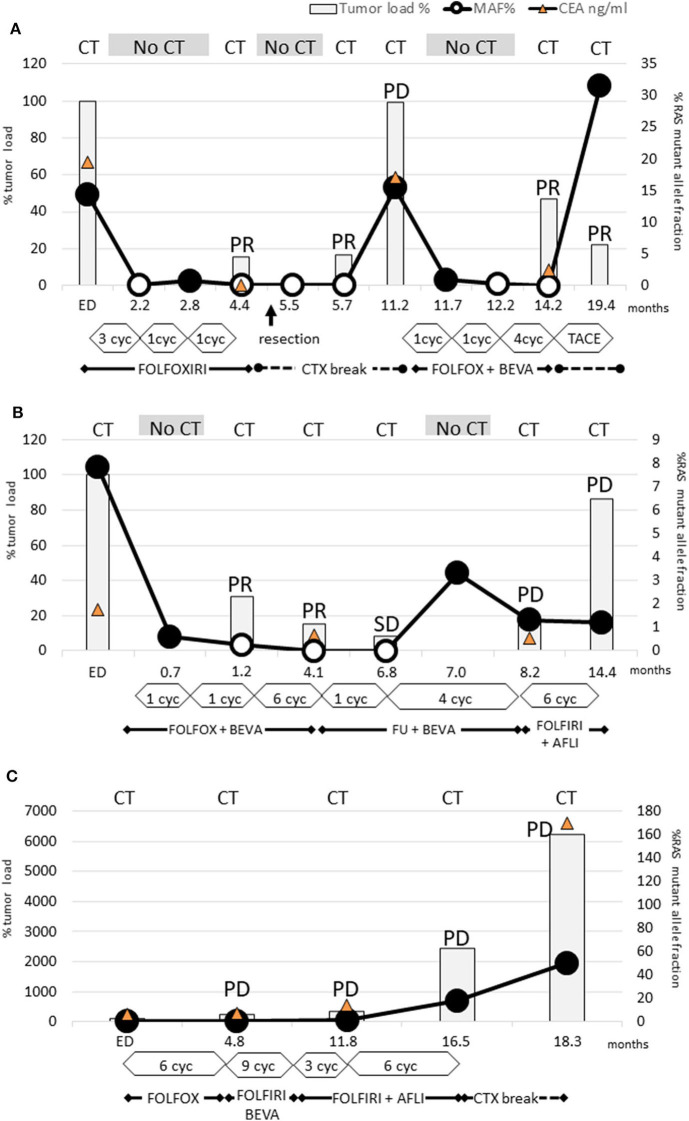
Dynamics of KRAS mutant clones measured in plasma samples. **(A)** *Patient 3:* RAS mutations disappeared already after 3 cycles FOLFOXIRI and remained not detectable for 9 months. PR after 6 cycles FOLFOXIRI made the patient eligible for tumor resection (arrow). After the following chemotherapy break PD occurred simultaneously with renewed rise of RAS mutation load. Already after 1 cycle FOLFIRI and bevacizumab the RAS mutations disappeared again and rose only after the next chemotherapy break due to TACE treatment. This case conclusively demonstrated that even multiple conversions of RAS status are possible with appropriate chemotherapy. **(B)** *Patient 4:* Already after 2 cycles FOLFIRI + bevacizumab the RAS mutations disappeared and remained not detectable for 6 months. Due to PR after 8 further cycles, the treatment was deescalated to 5-FU and bevacizumab. During this period, RAS mutation load increased again. Next, PD was diagnosed and the subsequent treatment change to FOLFIRI and aflibercept failed to achieve tumor response. However, RAS mutation load did not increase. This case points out that a renewed rise of RAS mutation load may be an early indicator for a lack of response. Furthermore, it can decrease during PD indicating that PD is caused by a RAS wild-type clone. **(C)** *Patient 12:* Neither FOLFOX nor FOLFIRI or addition of anti-VEGF antibody therapy led to a decrease of RAS mutation load or tumor response.

**Figure 2:**
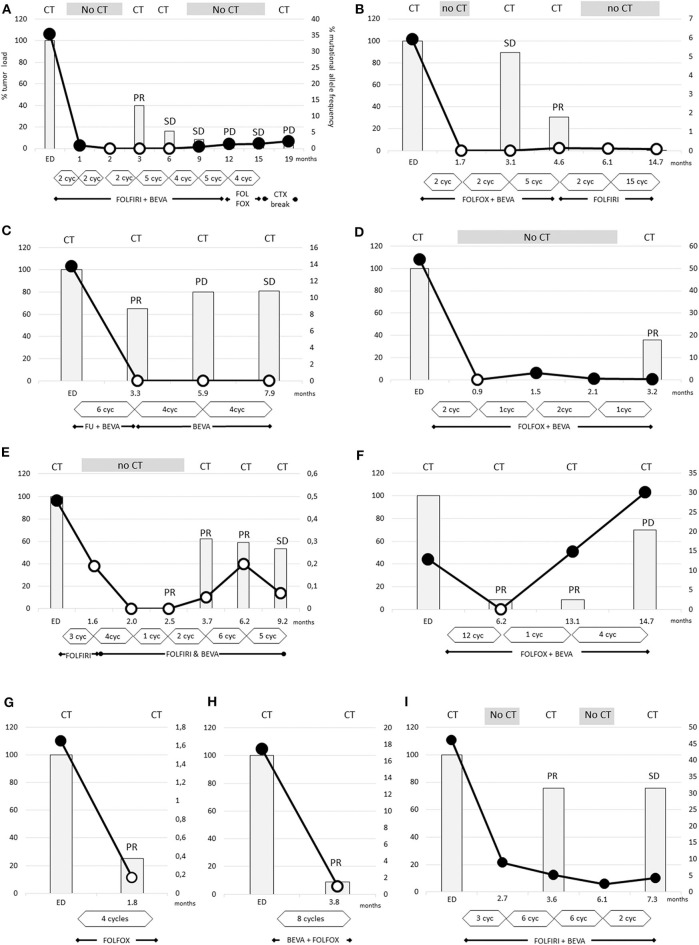
The dramatic decrease of RAS mutational load was detectable after 2 cycles **(A,B,D)** and the disappearance of RAS mutations occurs earliest after 2 cycles of therapy (B,D). In other cases **(A,C,E–H)** the disappearance is obvious after 4–12 cycles of therapy. The tumor loads, measured by CT scans, were strongly reduced by 75–90% in two cases **(F–H)**, and more moderately reduced by 20–60% in other cases **(A–E)**. In case **(I)**, despite the dramatic decrease of RAS mutational load the Ras mutations remained detectable. **(A)** Patient 7, **(B)** patient 8, **(C)** patient 2, **(D)** patient 6, **(E)** patient 10, **(F)** patient 9, **(G)** patient 1, **(H)** patient 5, and **(I)** patient 11.

Altogether, 69 samples were analyzed by liquid biopsy. In all patients with PR or SD significant decreases of RAS mutational loads were detected in plasma samples (Figures 1, 2, Table 1). In ten patients (91%), the ctDNA-based RAS mutational load decreased to a mutational allele frequency (MAF) below the cut-off of ddPCR (<0.25%) which was interpreted as conversion to RAS wild-type in ddPCR (Fisher's exact test *p* < 0.0001).

In nine cases, conversion to wild-type RAS (with a cut off level of < 0.02%) could be confirmed in the more sensitive OncoBEAM testing. Notably, new RAS mutations were not detectable by OncoBEAM testing. Definition of wild-type or mutated RAS status depends on the detection method's cut-off level, and this level was dependent on the amount of wild-type DNA (Supplementary Figures 3, 4). Patient 11, the only patient with PR without a conversion of RAS status, exhibited an extremely high proportion of RAS-mutated ctDNA at diagnosis of 46%. After 6 cycles FOLFIRI with bevacizumab the RAS mutational load declined by ~81%, however, mutated RAS ctDNA was still detectable (Figure 2I).

It is noteworthy that two patients converted to RAS wild-type after only 2 cycles of chemotherapy (Figures 2A,B). In all other patients, significant decreases of mutational frequencies were detected after about 4-5 cycles of therapy (range 1–12 cycles). There were no differences in the time span between bevacizumab-treated and -untreated patients regarding conversion into non-mutated RAS status (5.1 ± 1.4 vs. 3.3 ± 0.3 cycles; not significant different *p*-value:0.266). The RAS wild-type status remained unchanged during 3 weeks to more than 8 months.

No associations of the initial MAF level of RAS and the time (cycles) to reach RAS wild-type status or with the period of new RAS wild-type status were observed (see Supplementary Figure 7). Also, there were no association between the sidedness of the tumor and the reversion time points or period of RAS wild type status (*p*-value 0.955 and *p*-value 0.344). (It has to be considered that the examination of time points were biased by different time points of the sample withdrawal and the various clinical management of patients and by the small number of patients analyzed.)

Interestingly, patient 3 experienced even two conversions: the initial response after treatment with FOLFOXIRI resulted in a first conversion to RAS wild-type (Figure 1A). Following withdrawal of therapy due to prolonged infection and tumor surgery, the disease progressed clinically in parallel to reconversion into RAS-mutated status. During subsequent chemotherapy (FOLFOX, bevacizumab) conversion to RAS wild-type could be observed. During the off-treatment period, the patient reconverted into RAS-mutated status again.

The RAS mutational load of the patient suffering from PD (Figure 1C) increased continuously during the complete course of disease.

Despite depletion of RAS-mutated clones only moderate tumor reductions (about 50% tumor mass reduction) were observed in 7 cases. In only three cases the tumor load was lower than 20% compared to baseline condition. Accordingly, statistical testing in respect to an association of RAS disappearance and tumor shrinkage was not statistically significant (p = 1.0). This finding underlines that RAS mutational load reduction is not a marker for therapeutic response.

Our data show that in some cases the wild-type circulating DNA amount in plasma decreased in addition to the mutated variants. In these three cases (patients 6, 7, 11) the mutant allele frequencies prior to therapy were 54, 35, and 46%, respectively, and, the tumors were probably dominated by KRAS mutant cell clones. In all other cases (7/10) we observed that the amount of wild-type circulating DNA in plasma was stable or increased at the time point of RAS wild-type conversion (Supplementary Figures 3, 4). Three cases showed increasing wild-type circulating DNA amounts (Supplementary Figures 4B,H,I). In these cases, the decrease of RAS mutant fraction is in fact caused by the decrease of mutant RAS copies, and additionally by increase of wild type circDNA.

Neither BRAF V600E mutations nor PIK3CA mutations, which had not been found in patients' FFPE tissues, were detected in plasma samples during diagnosis and during the subsequent mCRC course.

In order to exclude false positive results of liquid biopsy derived from RAS mutations harbored in clonal hematopoiesis, genomic DNA isolated from peripheral blood cells was also investigated. These results clearly demonstrated that RAS mutations did not originate from the peripheral blood cells in our patients (Supplementary Figure 5).

### Methylated WIF1 Promotor ctDNA in Plasma

**Figure 3:**
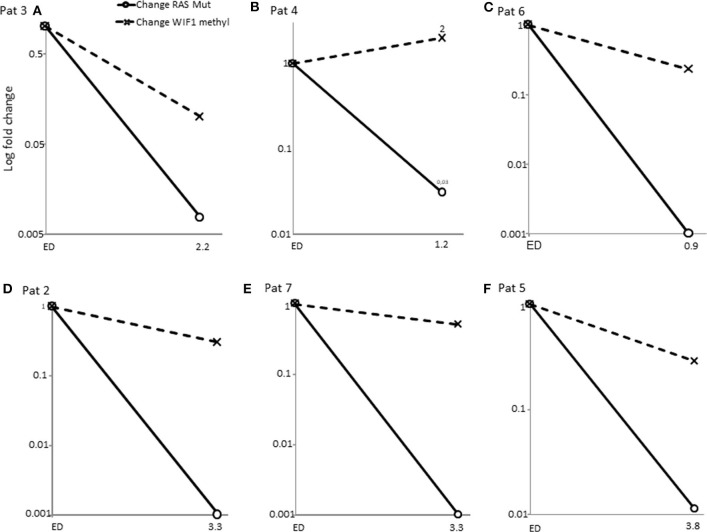
Change of WIF1 promotor methylation proportion vs. change of RAS mutational allele frequency. Samples of patients at the diagnosis time points vs. samples of the time point of disappearance of RAS mutations were measured by methylation specific ddPCR (see Supplementary File). WIF1 promotor methylation proportion remained detectable in samples with massive RAS MAF% reduction. **(A–F)** (solid line with circle, change of RAS mutant allele frequency; dashed line with cross, change of WIF1 promotor methylation proportion).

Promotor hypermethylation in CpG islands is a common epigenetic alteration in colorectal cancer and has been described in 70–90% of tumors (19). By means of WIF1-promotor methylation analyses of plasma derived circulating DNA we examined plasma samples of 8 patients. In Figure 3 the WIF1 methylated change of the samples of the diagnosis time point or earliest available time point were shown vs. the samples of the earliest available time points after RAS massive reduction. The WIF1 methylation proportion decreased in 6 cases but remained detectable or increased as compared to RAS mutational allele frequency (Figure 3B and Supplementary Table 2). Samples from three patients were not available. Thus, WIF1-promoter methylation assays confirmed the presence of circulating tumor DNA in 60% of mCRC with disappearance of RAS mutations during treatment.

## Discussion

We demonstrated that RAS mutations rapidly disappeared in patients after the first cycles of chemotherapy without significant tumor mass reduction. This occurred in all patients with PR or SD independently of the type and intensity of chemotherapy. The patient with PD did not show any decrease of RAS mutational load. These result in the Novel conclusion that CRC with RAS mutations at diagnosis are convertible into wild-type status after only a short time of anti-neoplastic treatment.

By now, detection of RAS mutations is essential for treatment management of patients with mCRC. These mutations result in constitutive activation of the GTPase KRAS, leading to permanent activation of downstream signaling of the EGFR (24, 25). Since 2013, RAS mutations have been considered the most important predictive biomarker of resistance against anti-EGFR therapy in mCRC, and remains the only straightforward predictive marker approved for clinical use. RAS gene testing has therefore become an essential part of the workup of CRC patients (26). Until today, due to the identification of other predictive KRAS and NRAS mutations, the prevalence of RAS-mutated mCRC at diagnosis raised to about 55% (5, 26). Currently, this means that for most patients treatment with anti-EGFR mAb is not taken into consideration.

Thus, it is important to elucidate the question whether patients with initially diagnosed RAS-mutated tumors retain this status during the complete disease course or whether RAS-mutated tumor cells are negative selected following chemotherapy and the tumor subsequently turns into a wild-type status, which could be potentially treated with anti-EGFR agents. Until today, this issue has not been explicitly addressed, yet, because repeated mutation analyses requiring sequential tumor biopsies during CRC course are infeasible.

The meanwhile available diagnostic tool of liquid biopsy in serially collected blood samples is predestined to answer the question of dynamic changes of RAS mutational status during first line therapy and to non-invasively analyze potential clonal selection of tumor cells over time.

Liquid biopsy (i.e., blood-based mutation analysis of cell-free circulating tumor DNA) is well-established in specialized clinical laboratories (16, 27) and allows for real-time characterization and monitoring of tumor heterogeneity as well as of treatment-related dynamic changes of molecular profiles of the overall disease regardless of tumor location (6, 16).

In our study, we combined the two most sensitive methods of liquid biopsy: BEAMing and ddPCR. First, ddPCR was used to detect RAS mutations. In samples with no detection of RAS mutations by ddPCR, the more sensitive BEAMing RAS assay was exploited to analyze the decrease of RAS mutations more precisely and to check upcoming new RAS mutations under treatment (17).

An important finding of our study is the accurate determination of the kinetics of RAS decay in blood in heretofore treatment-naive patients, which had so far remained poorly defined. At this point, our study essentially differs from Gazzaniga et al. and Raimondi et al., who published the switch of RAS mutated status to wild-type in 4 of 11 patients at a much later time point, during progressive disease (28–30). Furthermore, another varying and limiting factor of mentioned studies is that the measurements were performed with the less sensitive Idylla™ system (30, 31). The authors confirmed the RAS wild-type results by performing NGS analyses and found no RAS mutations, but new somatic mutations in one of the 4 patients and additionally measured germline mutations in each patient (30).

Our results are consistent with Sunakawa et al., who demonstrated conversion of RAS mutated to wild-type in plasma of 26/34 (76%) after 4 x FOLFOXIRI with bevacizumab (32). Spindler et al. reported about 27% (11/41) conversion rate from RAS mutated to wild-type at time point of progressive disease after 2nd line therapy, after exposure to fluoropyrimidine, oxaliplatin and irinotecan (33). Vidal et al. demonstrated decrease of RAS mutation load in patients with baseline RAS mutations responding to systemic therapy following 8–12 weeks of treatment (34). Measurements from an earlier point in time were not described in this work. The decrease of RAS mutation load was interpreted as an early predictor of response (34). Another interpretation appears to be more conclusive: Due to the fact that not a single patient achieved CT morphological complete remission during disappearance of RAS mutations, the CT tumor load contrasts RAS mutation load.

Thus, we are confident that the RAS mutation load in blood is not just another biomarker of tumor load, but rather a biomarker of negative clonal selection of RAS-mutated clones. It is significant to note that the fact that patients initially converted from RAS mutated to RAS wild-type did not show following increase of RAS mutation and remained RAS wild-type at time point of progressive disease [20% in our study; 69% in (32) and 55% in (33)] conclusively proves the negative clonal selection of RAS-mutated clones and contradicts the theory of lack of ctDNA shedding as the reason for absence of RAS-mutated ctDNA. We assume that the tumor is actually RAS wild-type during this period.

Hypermethylation of WIF1 CpG islands is considered to be a sensitive marker of colorectal cancer cells (21, 22). WIF1 promoter methylation analyses by ddPCR is a sufficient and sensitive tool to verify that DNA isolated from plasma derived from colorectal tumor cells. A deficiency of ctDNA release could be excluded by our results in 7 of 10 RAS mutation negative cases. Moati et al. (21) also used the WIF1 methylation assay. They found only two cases with WIF methylations out of eight RAS negative cases. Unfortunately, they didn't proof the primary samples of the RAS mutation cleared cases.

Accordingly, there are some tissue-based studies demonstrating decrease or loss of RAS mutations during chemotherapy. In the largest tissue-based study, Li et al. found significantly less RAS mutation in specimens after chemotherapy (46/105, 43.8%) as compared to specimens without chemotherapy (340/624, 54.5%) (*p* = 0.043) (8). Spindler et al. confirmed the liquid biopsy detected conversion from RAS-mutated to wild-type in tissue based RAS diagnostics of a metastasis (33).

Depending on the genetic context and type of RAS mutation, oncogenic RAS mutations can cause growth-promoting effects of mutated cell clones, or result in inhibition of differentiation and maintenance of stem cell properties (KRAS mutations), or in inhibition of apoptotic programs (NRAS mutations) (35). Based on our results, we conclude that antiproliferative chemotherapy could have a greater effect on KRAS-mutated cell clones with negative selection of mutated clones. In agreement with the disappearance of KRAS-mutated clones, the mutational allele frequency in plasma decreased.

As previously reported, NRAS mutations result in inhibition of apoptosis, and NRAS-mutated cells are therefore not directly affected by antiproliferative agents (35, 36). According to this observation, the eradication of NRAS-driven tumors by chemotherapy needs more time than the eradication of tumors driven by KRAS. Consequently, the mutational allele frequency of a patient with mutated NRAS (patient no. 9) decreased not until 12 cycles of CTX plus bevacizumab treatment were administered.

The depletion of mutant RAS alleles coincided with a reduction of tumor growth measured by CT scans. However, it should be noted that the remaining tumor load was over 40% from baseline at first follow-up in 5 of 11 cases. On the other hand, an increase of circDNA amount (wild type) in plasma could be measured (see Supplemental Material), and, simultanously the tumor load increased measured by CT scan. From this observation, we conclude that the depletion of KRAS mutant tumor cell clones can support the growth of wild-type clones.

Our data illustrate the multiclonal origin of CRC, its biological complexity and the probable interaction between tumor cell clones. Different models to explain tumor growth and development of resistance in CRC are currently under discussion. Tumor cell growth was adjusted under the negative selective pressure during therapy or, as described in other models, by omission of clonal interactions between tumor cells (37–40). To understand the clonal growth, molecular analyses of tissue biopsies of each metastasis would be necessary. These would, however, endanger the patients and therefore we avoided the performance of subsequent biopsies. On the other hand, the lack of data to RAS tissue status at the time point of RAS mutated allele disappearance in plasma is the main limitation to the interpretation of our data.

As we already know from anti-EGFR treated patients with initially RAS wild-type tumors, these patients acquire resistance to targeted treatment and RAS-mutated clones become detectable during progression. Omission of anti-EGFR therapy leads to rapid renewed decay of RAS-mutated clones and re-growth of the RAS wild-type cell population, which means a selection disadvantage for RAS-mutated cells (6, 9, 10). We previously described the influence of different therapies on RAS mutation load (6). Basically, the phenomenon that we are describing in this article is comparable with the decay of RAS-mutated clones in RAS wild-type mCRC patients at diagnosis that were initially treated with anti-EGFR mAb, developed RAS-mutated clones, and were switched to an anti-EGFR-free (and anti-VEGF mAb-containing) therapy. Thus, this process is a prerequisite for the feasibility of the “rechallenge-principle,” one of the currently most promising issues in personalized treatment of mCRC (6, 10, 14). A direct impact of anti-VEGF therapy on release of ctDNA has not been described yet.

Our findings provide new ideas in the stagnating development of therapies for RAS-mutated patients, which are the largest molecularly defined group of patients with mCRC. The exciting question is whether patients with RAS-mutated tumors at diagnosis and disappearance of RAS mutations in blood during therapy would benefit from treatment with anti-EGFR mAb analogous to RAS wild-type tumors at diagnosis. This concept would be especially promising for left-sided CRC, because patients with RAS wild-type left-sided CRC especially benefit from anti-EGFR treatment compared with anti-VEGF treatment when added to standard chemotherapy (41). With respect to potential clinical studies, it should be mentioned that the probability to harm patients with an anti-EGFR therapy without measurable RAS mutations is considered to be low. Data from the CRYSTAL study distinctly demonstrated that the efficacy of cetuximab inversely correlates with the RAS mutation frequency in tissues. Only patients with a RAS mutation frequency of more than 20% experienced a non-significant inferior PFS (42). The inverse correlation between the proportion of mutated DNA in tissue and the anti-EGFR therapy response rate was reported in numerous studies (43, 44). For example, Laurent-Puig et al. demonstrated that patients with <1% of mutant KRAS allele in tissue have equal PFS and OS than those with wild-type KRAS tumors. Currently, our study group is initiating a randomized phase II trial to investigate whether patients with left sided RAS-mutant mCRC at diagnosis will have a PFS benefit from addition of cetuximab to first-line therapy after RAS-mutation status has changed to wild-type during standard 1st-line treatment as monitored by longitudinal liquid biopsies (MoLiMoR-trial; EudraCT-No. 2019-003714-14 patient aim *n* = 144).

Furthermore, enrolling phase II and III clinical studies CONVERTIX and KAIROS trials will provide clues about the clinical significance of liquid biopsy-based conversion of RAS mutant mCRC into RAS wild-type (28, 45).

To our knowledge this is the first study that demonstrated a liquid biopsy-based conversion of RAS mutant mCRC into RAS wild-type early after the first cycles of chemotherapy combined with accurate determination of the kinetics of RAS decay. To prove a clinical benefit of anti-EGFR therapy for patients who converted to RAS wild-type, a prospective, randomized clinical trial is required. The precise determination of the kinetics of RAS decay in blood is crucial for future studies exploiting ctDNA kinetics to guide therapy in this setting.

## Data Availability Statement

All datasets generated for this study are included in the article/Supplementary Material.

## Ethics Statement

The studies involving human participants were reviewed and approved by The University of Bochum Ethical Committee registration number: 16-5961. The patients/participants provided their written informed consent to participate in this study.

## Author Contributions

AB, SK-S, and RS: designed the research and wrote the paper. AB, SK-S, IW, RS, MP, TM, and SL: performed the measurements or analyzed the results. MM and YA-S: performed CT measurements. All authors: contributed to the article and approved the submitted version.

## Conflict of Interest

AB has received honoraria for lectures from Amgen, Merck Serono, Roche, Servier. The remaining authors declare that the research was conducted in the absence of any commercial or financial relationships that could be construed as a potential conflict of interest.

## Abbreviations

5-FU: 5-Fluorouacil
BEAMing: beads, emulsion, amplification magnetics
ctDNA: circulating cell-free tumor DNA
ddPCR: digital droplet PCR
EGFR: epidermal growth factor receptor
FOLFIRI: FOLinic acid, Fluorouacil, IRInotecan
FOLFOX: FOLinic acid, Fluorouacil, Oxaliplatin
FOLFOXIRI: FOLinic acid, Fluorouacil, OXaliplatinm IRInotecan
MAF: mutational allele frequency
mCRC: metastatic colorectal cancer
OS: overall survival
PD: progressive disease
PFS: progressive free survival
PR: partial remission
SD: stable disease
VEGF: vascular endothelial growth factor
