# Supplementary material for: Evolution of RAS Mutational Status in Liquid Biopsies During First-Line Chemotherapy for Metastatic Colorectal Cancer
Source: Front Oncol. 2020 Jul 16;10:1115. doi: 10.3389/fonc.2020.01115 (PMC7378792; doi:10.3389/fonc.2020.01115)
Supplement: Supplementary file 1 [file Presentation_1.pdf]

Supplementary figure 1

A

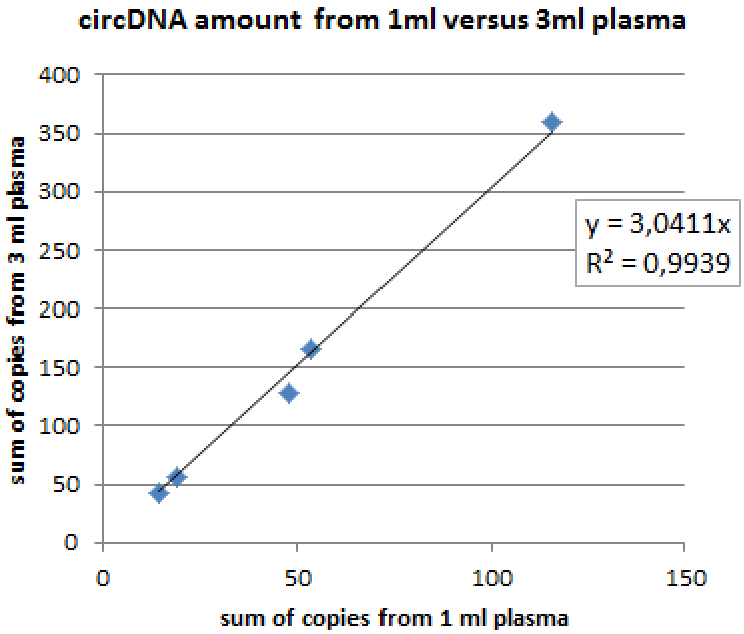

B

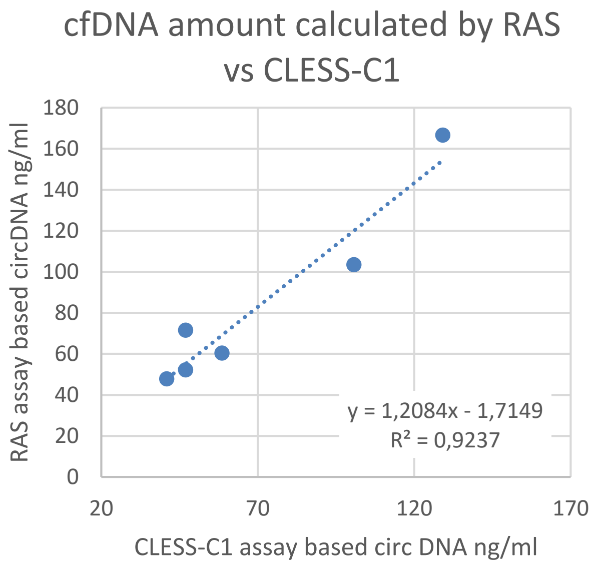

C

#Pat	minimal value of sum of copies	hapl human genome equivalents	ng
12	1017,8	20285	67
6	483,8	9643	32
10	389,2	7757	26
3	382,2	7617	25
5	331,8	6613	22
7	274,4	5469	18
11	188,2	3750	12
8	183,8	3662	12
1	124,5	2480	8
9	105,1	2095	7
4	101,9	2031	7
2	80,6	1607	5

Supplementary figure 1 A The amount of cfDNA isolated from 1 ml plasma and 3 ml plasma were compared. The data given in the supplementary figures 3 and 4 are normalized to 1 ml plasma.
B The amount of cfDNA were calculated by RAS assay measured numbers of copies versus CLESS-C1 measured number of copies. The both assays gave similar results regarding that the CLESS-C1 assay included bisulfid conversion step of cfDNA. **C** The minimal value of sum of copies in assays were converted to human haploid genome equivalents and to ng (by 3.3 pg per haploid genome equivalent). The theoretical limit of detection in the lowest case of patient 2 was 0,06%.

supplementary figure 2

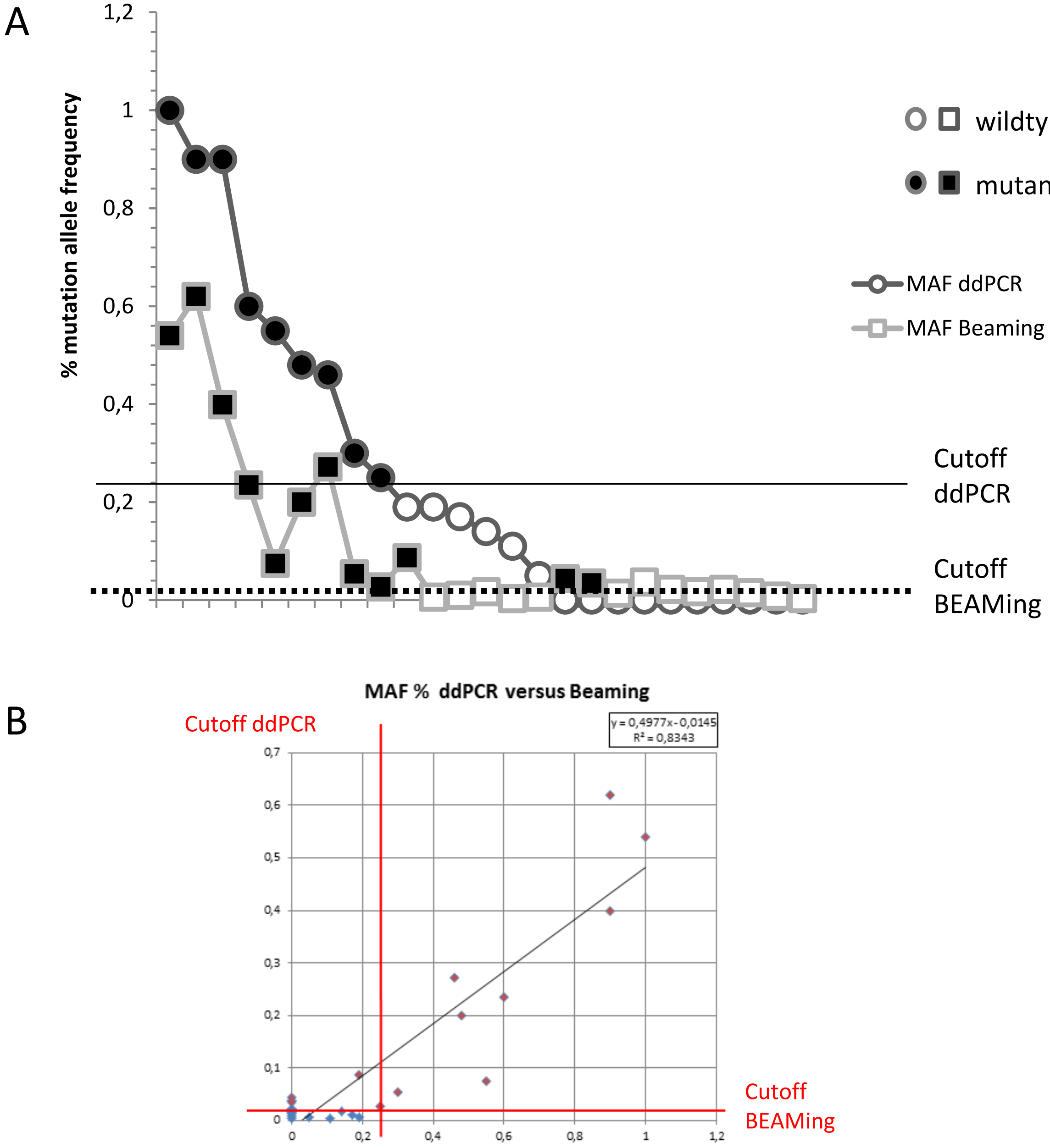

supplementary figure 2

Comparison of KRAS mutation screening by ddPCR and by ONCOBEAM RAS IVD analysis.
The ddPCR input DNAamount followed the recommendation of Bio-Rad manual and https://doi.org/10.1007/978-1-4939-7778-9.

A) MAF% values of ddPCR and BEAming under 1.2% were shown. Three samples (3/25) measured by ddPCR under cutoff level were determined as mutated by the ONCOBEAM RAS analysis.

B) The comparison of ddPCR and BEAming including MAF% values lower than 1.2% show a moderate agreement with a correlation coefficient of R^2=0,834, and the comparison including **all** MAF% values of ddPCR versus BEAming gave an excellent agreement with a correlation coefficient of R^2=0,9998. The limit of detection of BEAming procedure was determined at 0.02 % MAF as described in Diehl F, Li M, He Y et al. „BEAming: single-molecule PCR on microparticles in water-in-oil emulsions”. Nat Methods 2006; 3: 551–559

Supplementary Table 1

supplementary information to the ddPCR assays purchased from Bio-Rad Laboratories Inc. as recommended by the MIQE criteria according to Huggett JF, Foy CA, Benes V, Emslie K, Garson JA, Haynes R, Hellemans J, Kubista M, Mueller RD, Nolan T, Pfaffl MW, Shipley GL, Vandesompele J, Wittwer CT, Bustin SA. The Digital MIQE Guidelines: *M*inimum *I*nformation for Publication of *Q*uantitative Digital PCR *E*xperiments Clin Chem. 2013 Jun;59(6):892-902. doi: 10.1373/clinchem.2013.206375.

Gen name and Mutation	RefSeq Accession	Unique assay ID	Amplicon length	Context Sequence
PIK3CA c.1633G>A p.E545K	NM_006218.2	dHsaCP2000075	80nt	GAGATCCTCTCTCTGAAATCACTGAG<br>CAGGAGAAAGATTTTCTATGGAGTC<br>ACAGGTAAGTGCTAAAATGGAGATT<br>CTCTGTTTCTTTTTCTTTATTACAGAA<br>AAAATAACTGAATTTGGCTG
PIK3CA wild type	NM_006218.2	dHsaCP2000076	75nt	
PIK3CA c.3140A>G p.H1047R	NM_006218	dHsaCP2000077	80nt	GCCTTAGATAAAACTGAGCAAGAGG<br>CTTTGGAGTATTTCATGAAACAAATG<br>AATGATGCAC[A/G]TCATGGTGGCTG<br>GACAACAAAAATGGATTGGATCTTCC<br>ACACAATTAAACAGCATGCATTG
PIK3CA wild type for p.H1047R	NM_006218	dHsaCP2000078	80nt	
BRAF c.1799T>A, p.V600E	NM_004333.4	dHsaCP2000027	91nt	CACTCCATCGAGATTTCACTGTAGCT<br>AGACCAAAATCACCTATTTTTACTGT<br>GAGGTCTTCATGAAGAAATATATCTG<br>AGGTGTAGTAAGTAAAGGAAAACAG<br>TAGATCTCATTTTCCTATCA
BRAF wild type	NM_004333.4	dHsaCP2000028	91nt	
KRAS G12/G13 Screening Kit #1863506 G12D, G12A, G12C, G12D, G12R, G12S, G12V, G13D	NM_004985,NM_033360	dHsaMDV2510586	57nt	TTATTTTTATTATAAGGCCTGCTGAAA<br>ATGACTGAATATAAACTTGTGGTAGT<br>TGGAGCTG[G/C]TGGCGTAGGCAAG<br>AGTGCCTTGACGATACAGCTAATTCA<br>GAATCATTTTGTGGACGAATAT

Primers were purchased from Bio-RAD laboratories:

WIF1 meth Forward GGTTGTGTATTTATAGTGCGGTG
reverse ATCTCTAAATACCCTTCTCCGAA
Probe FAM-GAAGTGGG CGTGTAGGGTTGGCG-MGBNFQ

C-LESS-C1 Forward TTGTATGTATGTGAGTGTGGGAGAGAGA
Reverse TTTCTTCCACCCCTTCTCTTCC
Probe HEX-CCTCCCCCTCTAACTCTAT MGBNFQ

supplementary figure 3

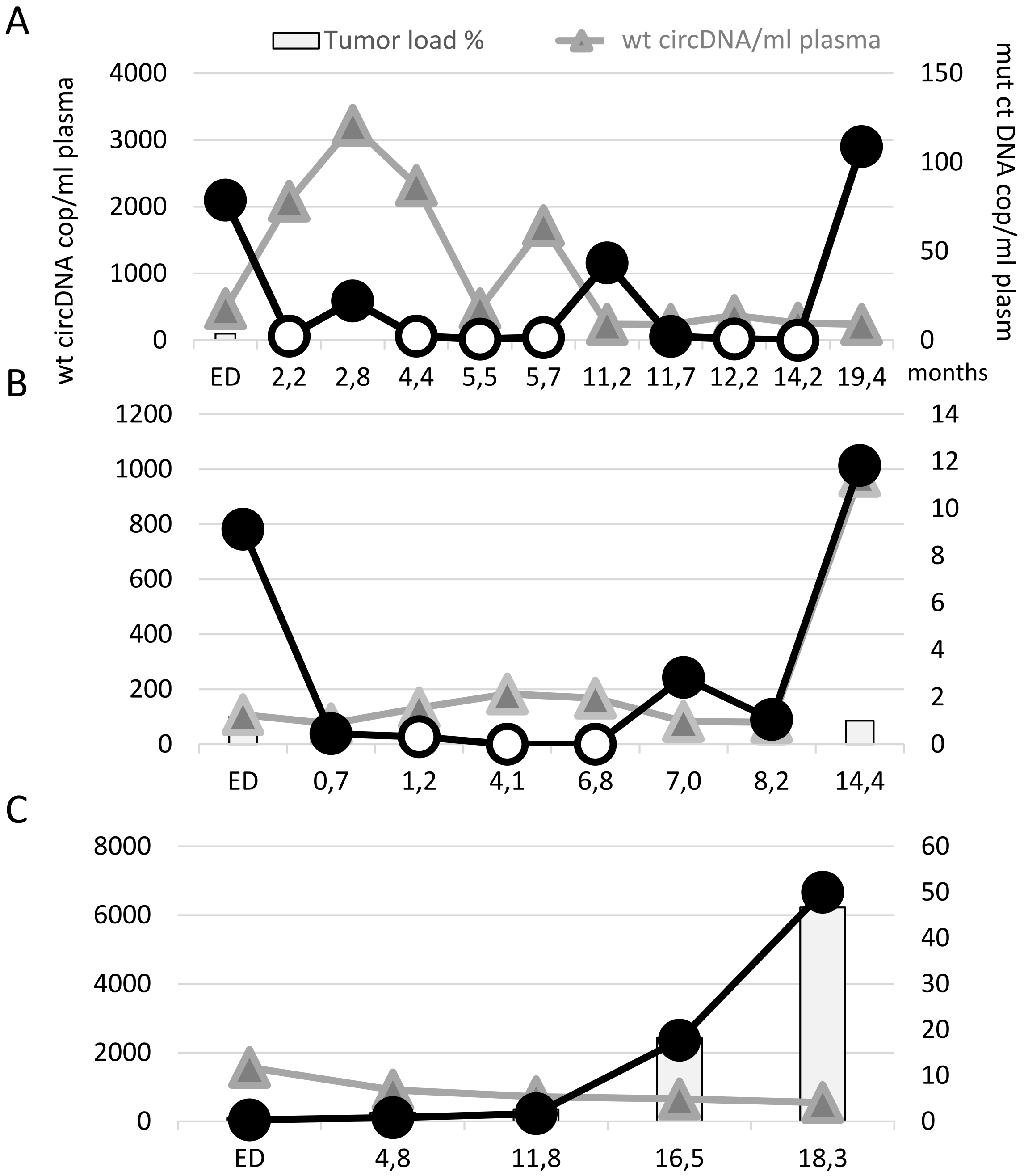

supplementary figure 3 Dynamic changes in the KRAS wild type and mutated copies normalized to 1 ml of plasma that were measured during the treatment of sensitive (A,B) and therapy-resistant (C) patients. Circles, FAM labeled RAS mutant copies, Triangles HEX labeled RAS wild type copies; all data normalized to 1 ml plasma.

In response to therapy, the wild type ctDNA amount was increased, whereas the absolute amount of mutational allele was dropped down after 3 cycles of therapy (2.2 months). The MAF % reduction (figure 1 A) was a result of both changes (A Patient 3). Whereas the wild type ctDNA amount remained more or less stable , the absolute amount of mutated ctDNA was dramaticallly reduced in the first therapy cycle and further decreased under the cut off during therapy . The wild type ctDNA amount was only moderately changed. The amount of mutated ctDNA increased again during the following therapy cycles and supplementaryly the wild type ctDNA amount increased when tumor progression occurs (B Patient 4). In the plasma of the therapy resistant patient with progression disease no reduction of absolute amount of mutated ctDNA could be detected. The wild type ctDNA amount remained stable or moderaly decreased (C Patient 12)

supplementary Figure 4

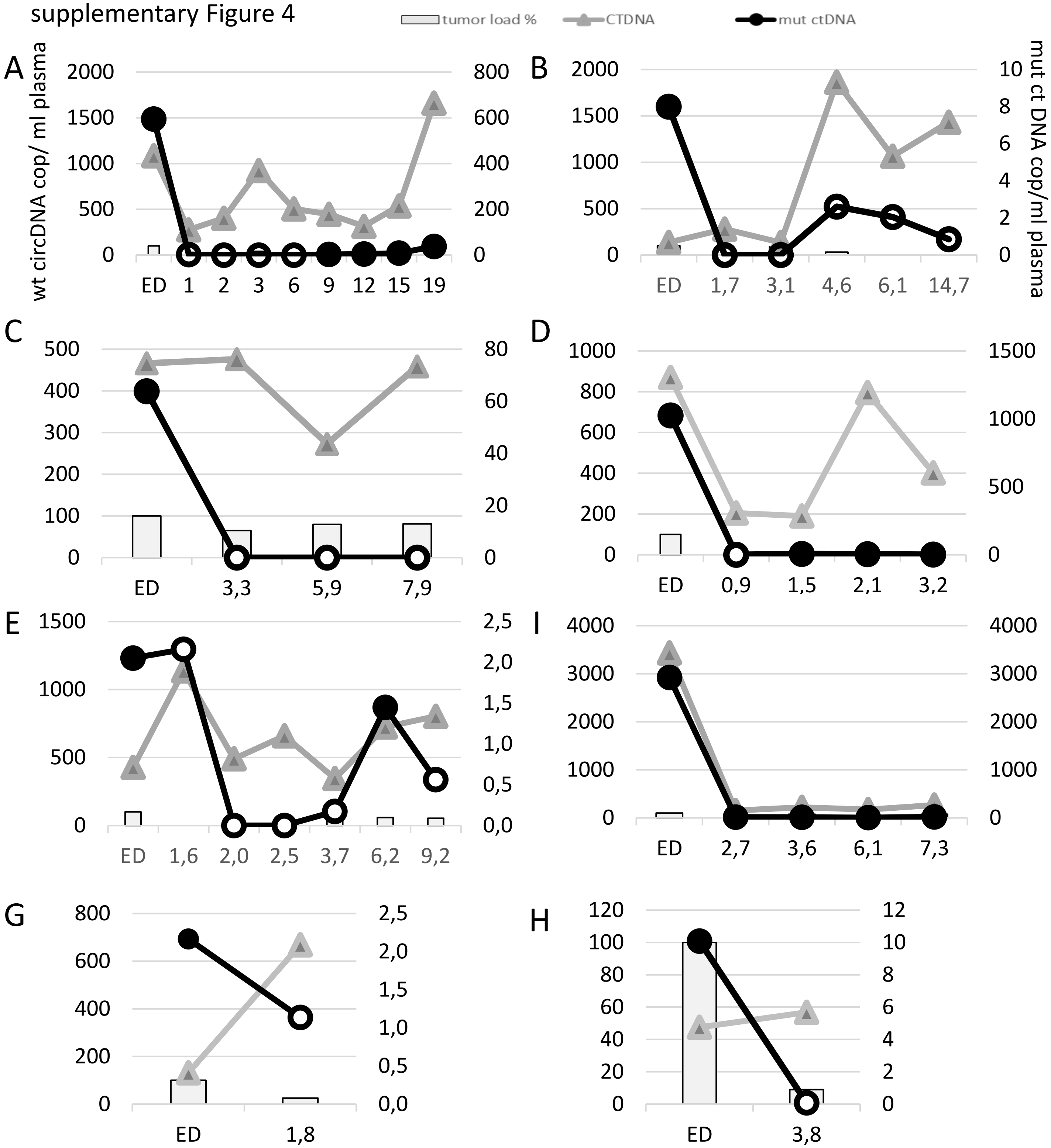

Supplementary figure 4: Changes of Ras mutated ctDNA and wild type ctDNA during therapy. The decrease of absolute amounts of mutated KRAS ctDNA (normalized to 1 ml plasma) was obvious in all 8 cases. In three cases the drop down of mutated ctDNA is accompanied by a decrease of wildtype ctDNA (A pat7,D pat 6, I pat 11). In 5 cases the wt ctDNA remained stable or increased in contrast to the mutated ctDNA amount (B pat 8, C pat 2, E pat 10, G pat1, I pat 11). Circles, FAM labeled RAS mutant copies; Triangles, HEX labeled RAS wild type copies; all data normalized to 1 ml plasma.

supplementary figure 5

Analysis of hematopoeitic origin of Ras mutated allels in circDNA illustrated by comparison of genomic DNAs and circDNA MAF%

Patient	ctDNA RAS Mut MAF [%]	gDNA cellular RAS Mut MAF [%]
6	57,1	0,1
11	46	0,36
7	35,4	0,12
5	17,5	0,07
3	14,3	0,04
2	13,7	0,07
9	12,8	0,02
4	7,8	0
8	5,9	0,07
12	0,8	0,01
10	0,48	0,07
1	0,17*	0,12*

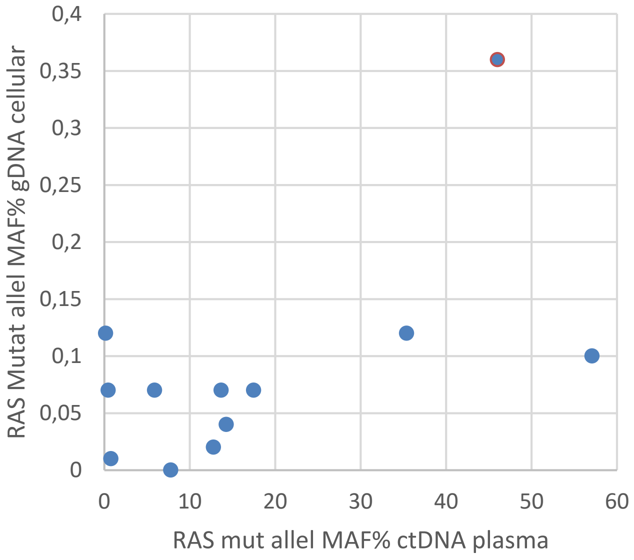

Supplementary figure 5

Analysis of hematopoeitic origin of Ras mutated allele fragments in circDNA illustrated by comparison of genomic DNAs and circDNA MAF%. RAS allele analyses were done with minimal 27-100 ng input DNA by ddPCR.

Eleven of the cellular DNA sample showed no significant amounts of mutated allele fragments. In one sample (patient 11) the mutated allele fragments could be measured with 0.36 % MAF. In the corresponding circulating plasma DNA the RAS MAF % was determined with 46%. Prior to isolation the genomic DNA using Qiagen DNA blood kit the plasma were carefully removed but no blood cell separation was done. We interpret the 0.36% MAF in this cellular DNA sample as a contamination by high amounts of mutated DNA fragments in plasma.

*Initial samples are not available. These data originated from the sample 1.8 months after therapy.

supplementary figure 6

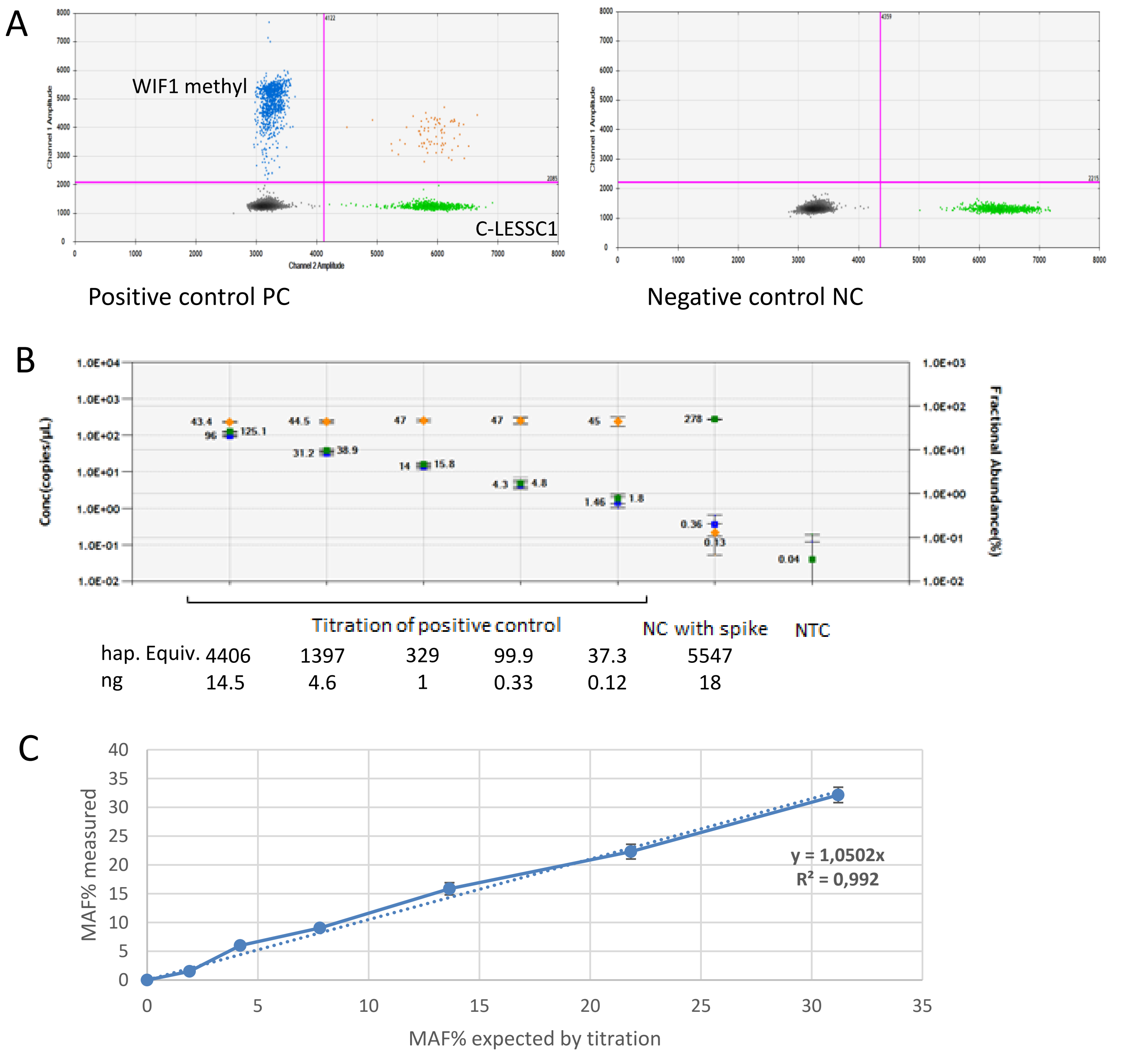

Supplementary figure 6: Methylation specific ddPCR of WIF1 promotor CpG islands versus C-LESS-C1

Two dimensional blots of samples of ddPCR to detect WIF1 promotor methylation (**A**). Primers and ddPCR conditions of methylation specific assay were designed according to the procedure described in Yu M et al. 2015 Epigenetics 10:9, 803-809, Roperch et al. 2013. Positive control DNA amount was titrated to check influence of input DNA on WIF1 promotor methylation assay **(B)** and mixtures of defined positive and negative samples were measured (**C**). Green, HEX copies of CLESS-C1; blue, FAM copies of methylated WIF1 promotor; orange, % fractional abundance determined by FAM copies/sum of HEX&FAM.

Unmethylated EpiTect control DNA (NC) purchased from QIAGEN (Cat #59665), and methylated human DNA (PC) purchased from Zymo Research (Cat. D5011).

supplementary Table 2 **WIF1 methylation change versus RAS mutational change of patients**

Patient	months after ED	change RAS	change WIF1 methyl	MAF% WIF1 Methyl	wif1 methyl cop/ml plasma	CLESSC1 cop/ml plasm
#1	ED	1	not analyzed			
	1,7	0,103				
#2	ED	1	1	12,8	14,8	101,9
	3,3	0,0	0,3	3,9	1,5	97,0
#3	ED	1	1	14,57	75,2	440,7
	2,2	0,008	0,1	1,00	18,1	1788,9
#4	ED	1	1	0,82	0,4	44,1
	1,23	0,03	2	1,64	1,1	65,3
#5	ED	1	1	10,2	35,3	310,1
	3,8	0,011	0,288	2,9	*1,0*	34,2
#6	ED	1	1	10,30	578,7	5055,6
	0,9	0	0,23	2,40	4,0	166,4
#7	ED	1	1	10,00	305,9	2758,5
	3,27	0	0,51	5,10	12,4	230,2
#8	ED	1	not analyzed			
	1,7	0				
#9	6,2	1	0	0	0,9	0,88
	15,7	7526	pos	0,4	14,5	3956,30
#10	ED	1	1	22,6	171,1	585,9
	15,1	0	0,049	1,1	14,5	1280,7
#11	ED	1	not analyzed			
	1,7	0,190				
#12	ED	1	1	2	31,1	1533,8
	4,77	2,67	0,63	1,26	11,41	891,85
	11,8	5,67	1,22	2,43	15,56	623,78

Supplementary figure 7 **Additional information to statistical analyses**

No association between RAS MAF % and time to reach RAS wild-type or periode of RAS – wild-type state

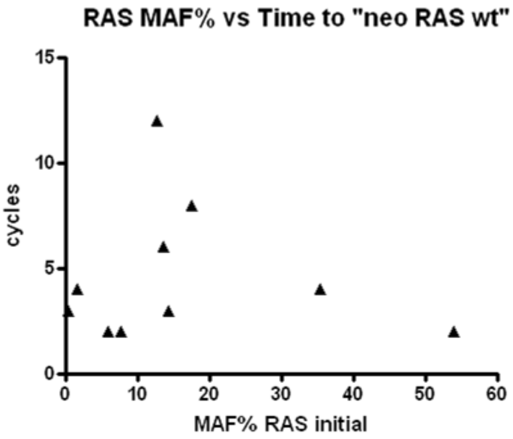

No associatien of RAS MAF initial value and the periode of RAS wild-type status > 4 cycles or <= 4 cycles

cycles to neo ras <=4	cycles to neo ras >4
14,3	13,7
12,8	17,5
1,65	12,8
5,9	
7,8	
35,4	
54	

Unpaired t-test p-value 0.59

Comparison of left or right sided type of tumors and therapy cycles to wild-type status of RAS

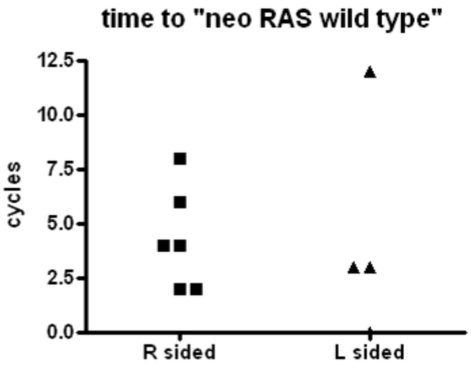

Unpaired t test with Welch's correction
P value 0,9558

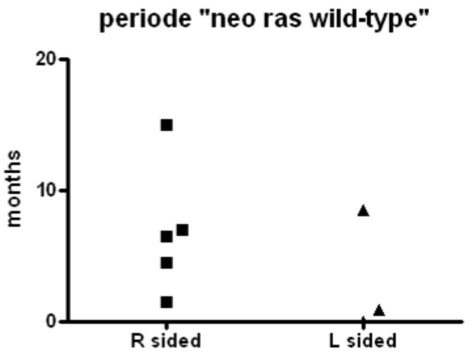

Unpaired t test with Welch's correction
P value 0,344
